# First-principles calculations of structural, electronic, magnetic and elastic properties of Mo_2_FeB_2_ under high pressure

**DOI:** 10.1098/rsos.172247

**Published:** 2018-07-18

**Authors:** Bin Wang, Benyuan Ma, Wei Song, Zhe Fu, Zhansheng Lu

**Affiliations:** 1 Physics and Electronic Engineering Department, Xinxiang University, Xinxiang 453003, People's Republic of China; 2 College of Physics and Electronic Engineering, Nanyang Normal University, Nanyang 473061, People's Republic of China; 3 College of Physics and Materials Science, Henan Normal University, Xinxiang 453000, People's Republic of China

**Keywords:** high pressure, electronic properties, magnetism, elastic properties

## Abstract

The structural, electronic, magnetic and elastic properties of Mo_2_FeB_2_ under high pressure have been investigated with first-principles calculations. Furthermore, the thermal dynamic properties of Mo_2_FeB_2_ were also studied with the quasi-harmonic Debye model. The volume of Mo_2_FeB_2_ decreases with the increase in pressure. Using the analysis of the density of the states, atom population and Mulliken overlap population, it is observed that as the pressure increases, the B–B bonds are strengthened and the B–Mo covalency decreases. Moreover, for all pressures, Mo_2_FeB_2_ is detected in the anti-ferromagnetic phase and the magnetic moments decrease with the increase in pressure. The calculated bulk modulus, shear modulus, Young's modulus, Poisson's ratio and universal anisotropy index all increase with the increase in pressure. From thermal expansion coefficient analysis, it is found that Mo_2_FeB_2_ shows good volume invariance under high pressure and temperature. The examination of the dependence of heat capacity on the temperature and pressure shows that heat capacity is more sensitive to temperature than to pressure.

## 1. Introduction

Mo_2_FeB_2_ is widely used as a wear-resistant material owing to its high degree of hardness, high melting point and high electrical conductivity [1]. Current studies to improve the mechanical properties of Mo_2_FeB_2_ mainly introduce Mn, Nb, V, Cr, Ni and C [2–7]. Mn addition can improve the wettability of the Fe binder phase on the Mo_2_FeB_2_ hard phase. This enhancement is observed because Mn can refine the grains, decrease the porosity and increase the phase uniformity of Mo_2_FeB_2_ [2,6]. Addition of V and Nb can also refine the grains [3,7]. Furthermore, with the increase in the Nb/V content, the hardness and transverse rupture strength are both first enhanced and then decreased [7]. Additions of Cr and Ni enhance the hardness and transverse rupture strength [4]. Addition of carbon can improve the hardness but it decreases the transverse rupture and fracture toughness [5].

Theoretical studies of Mo_2_FeB_2_ are rare. Using empirical electron theory of solids and molecules, Pang *et al*. predicted that the brittleness of Mo_2_FeB_2_ arises from weak bonds [8]. He *et al*. found that Mo_2_FeB_2_ exhibits the largest shear and Young's moduli (*E*) due to its strong chemical bonding among the Mo_2_XB_2_ and MoX_2_B_4_ (X = Fe, Co, Ni) ternary borides with first-principles methods [9]. By the first-principles method, it is also found that addition of Cr can improve the volume deformation resistance of Mo_2_FeB_2_. Addition of Mn can improve the shear deformation resistance of Mo_2_FeB_2_ [10]. It is worth pointing out that the structure, electronic density of states (DOS), and magnetic and elastic properties of Mo_2_FeB_2_ under normal pressure have been studied by us before [11]. It was found that magnetism has a great impact on the crystal structure and mechanical properties. The anti-ferromagnetic (AF) case is the ground state. The B–B and B–Mo bonds play an important role in the shear modulus. The Fe atom contributes the most to the magnetism.

To date, there have been no reports on Mo_2_FeB_2_ behaviour under high pressure. On the one hand, high temperature and high pressure can help increase the density of the hard phase [12,13]. On the other hand, Mo_2_FeB_2_-based cermets are typically used in extreme conditions (high pressure and high temperature). The variation of the magnetic properties and structure of Mo_2_FeB_2_ under high pressure is still unknown. Magnetism will affect the accuracy of the calculation of the crystal structure. Thus, it is necessary to study the electronic structure, elastic properties, magnetic properties and thermodynamic properties of Mo_2_FeB_2_ under high pressure.

## 2. Calculation method and crystal structure

The calculation method in this paper was similar to that of previous work [11]. The work was conducted based on density functional theory [14,15] with the calculations performed using the Cambridge Serial Total Energy Package (CASTEP) plane wave code [16]. The interaction of the ionic core and valence electrons was modelled with ultrasoft pseudopotentials. The valence states considered here correspond to B 2s^2^2p^1^, Fe 3d^6^4s^2^ and Mo 4d^5^5s^1^. The generalized gradient approximation in the Perdew–Burke–Ernzerhof form is used to describe the exchange and correlation terms [17,18]. The integration over the Brillouin zone was performed with the Monkhorst and Pack *k*-point mesh integrations [19]. The cut-off energy was set to 330 eV, and the 5 × 5 × 8 *k*-point grid was used. The convergence conditions were set as the maximum force on the atom below 0.01 eVÅ^−1^, the maximum stress below 0.02 GPa and the maximum displacement between the cycles below 0.0005 Å. Hydrostatic pressure was applied in the *x*, *y* and *z* directions simultaneously with an increase of 10 GPa each time. Furthermore, the AF ground state was first set before the geometric optimization. The setting method can be found in [11]. Then, the lattice optimization with spin polarization was performed.

In this work, the unit cell that contains 4 Mo atoms, 2 Fe atoms and 4 B atoms with periodic boundary conditions was used. Mo_2_FeB_2_ with tetragonal symmetry belongs to the *P*4/mbm space group. Furthermore, the ground state of Mo_2_FeB_2_ is AF [11]. The calculated data listed in table 1 agree well with the previous theoretical and experimental results [11,20,21]. The largest error is less than 1.6% between the volume *V*_0_ obtained by our calculations and the experimental data of Gladyshevskii [20].

**Table 1.** Calculated equilibrium lattice parameters (*a* and *c*) of Mo_2_FeB_2_ compared with experimental data and theoretical data.

Mo_2_FeB_2_	*a*(Å)	*c*(Å)	*V*_0_(Å^3^)	*c*/*a*
present	5.748	3.157	104.317	0.549
theoretical [11]	5.743	3.159	104.19	0.550
experimental [20]	5.807	3.142	105.952	0.541
experimental [21]	5.782	3.148	105.242	0.544

## 3. Results and discussion

### 3.1. Crystal structures under pressure

**Figure 1.**
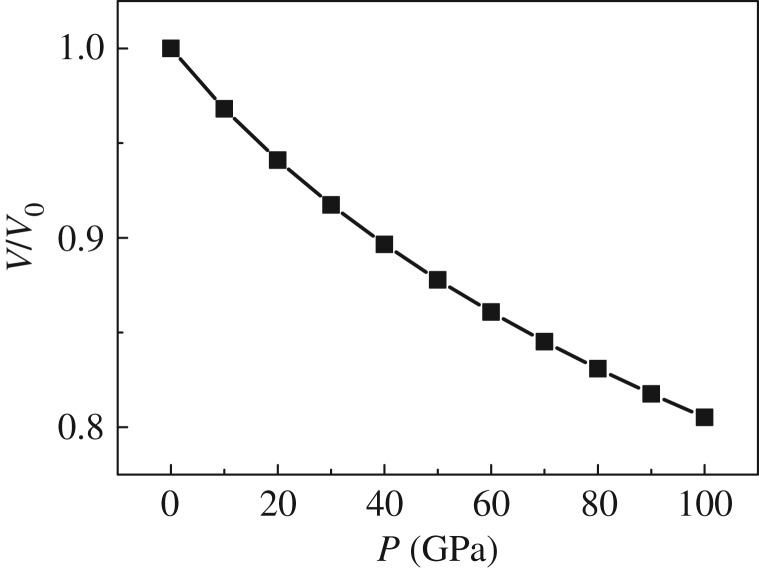
The relationship between normalized volume *V*/*V*_0_ and pressure for tetragonal Mo_2_FeB_2_.

According to the whole calculation, Mo_2_FeB_2_ maintained tetragonal symmetry and the *P*4/mbm space group. Furthermore, the atoms almost keep the same fractional coordinates, and the largest displacement (fractional coordinates) is less than 2% (supporting data). Figure 1 shows the relationship between the normalized volume *V*/*V*_0_ and the pressure, where *V*_0_ is the equilibrium volume at zero pressure. It can be observed that the volume decreases with increasing pressure.

### 3.2. Electronic structure and electronic population under pressure

**Figure 2.**
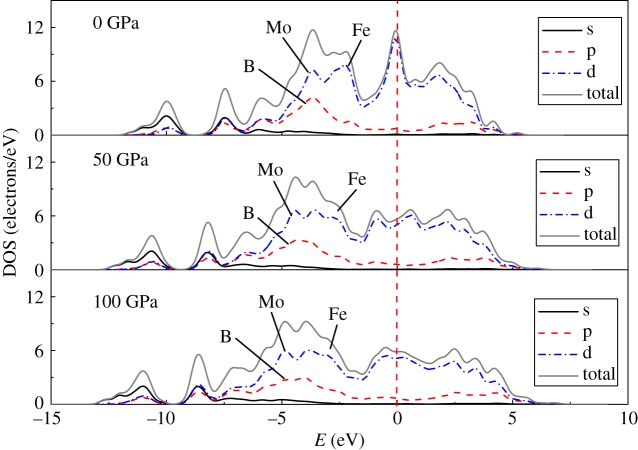
PDOS and TDOS of Mo_2_FeB_2_ under 0 GPa, 50 GPa and 100 GPa.

The calculated partial density of states (PDOS) and the total density of states (TDOS) under 0, 50 and 100 GPa around the Fermi level are shown in figure 2. Mo_2_FeB_2_ retained its metallic character at the Fermi level under different pressures. There are two peaks in the energy range from −15 to −7.5 eV that are composed of 2 s and 2p bands of B. This region of the DOS corresponds to the B–B covalent bonds composed of the strongly hybridized B s and p states and gives a positive contribution to the shear modulus. With the pressure increasing, the two peaks move to lower energy, which means that the B–B bonds are strengthened. The PDOS range from −7.5 eV to −2 eV is predominantly composed of B 2p, Mo 4d and Fe 3d bands. However, this region is mainly composed of the strongly hybridized B p and Mo d states (forming the B–Mo covalent bonds) and the less hybridized B p and Fe d states (forming the B–Fe covalent bonds). As the pressure increases, the energy range of this hybridized region increases, the B p and Fe d hybrids increase and the B–Mo covalency decreases. Around the Fermi level, the DOS are mainly the d bands of Mo and Fe, which form the Fe–Mo, Fe–Fe and Mo–Mo metal ion bonds, making a negative contribution to the shear modulus.

The electronic population can also be used to analyse the electronic structure and the covalent or ionic nature of a bond [22]. A high value of the bond population indicates the strong covalency of a bond. Otherwise, the bond is ionic. The results are listed in table 2, which can be separated into three categories of chemical interactions, namely, B–B, B–Mo(Fe) and Mo(Fe)–Mo(Fe) bonds. All bond lengths decrease as the pressure increases, which is due to the shrinking of the volume. The B–B and B–Fe bond populations increase with increasing pressure, which means that the covalence of the B–B and B–Fe bonds increases. On the other hand, the B–Mo I, B–Mo II, Fe–Mo, Mo–Mo I and Mo–Mo II bond populations decrease with increase in pressure. This finding indicated that the ionicity of the B–Mo I and B–Mo II bonds increases, and the Fe–Mo, Mo–Mo I and Mo–Mo II populations belong to the anti-bonding states. Furthermore, until the pressure reaches 100 GPa, the B–Fe and B–Mo I populations are almost the same. As the pressure increases, B–Mo II changed to anti-bonding. Overall, the B–B bond is the strongest covalent bond, which is in agreement with the results of the DOS analysis (strongly hybridized B s and p states).

**Table 2.** Mulliken overlap population analysis for bonds of Mo_2_FeB_2_ under different pressures.

	B–B		B–Fe		B–MoI		B–MoII		Fe–Mo		Mo–MoI		Mo–MoII	
*P* (GPa)	length	population	length	population	length	population	length	population	length	population	length	population	length	population
0	1.83	0.60	2.32	0.16	2.32	0.59	2.33	0.31	2.64	0.04	2.89	0.04	2.99	−1.05
10	1.82	0.61	2.29	0.18	2.30	0.58	2.30	0.27	2.61	−0.01	2.86	0.05	2.96	−1.18
20	1.80	0.61	2.27	0.19	2.27	0.56	2.28	0.23	2.59	−0.06	2.83	0.05	2.93	−1.30
30	1.80	0.62	2.25	0.20	2.26	0.55	2.28	0.21	2.58	−0.09	2.81	0.04	2.91	−1.36
40	1.78	0.62	2.22	0.22	2.23	0.51	2.25	0.16	2.55	−0.18	2.78	0.02	2.88	−1.55
50	1.77	0.63	2.21	0.24	2.22	0.49	2.24	0.12	2.53	−0.25	2.76	0.00	2.85	−1.66
60	1.76	0.63	2.19	0.26	2.20	0.46	2.23	0.09	2.51	−0.32	2.74	−0.03	2.83	−1.78
70	1.75	0.64	2.17	0.27	2.19	0.43	2.21	0.05	2.50	−0.39	2.73	−0.06	2.81	−1.90
80	1.75	0.65	2.16	0.29	2.17	0.40	2.20	0.01	2.48	−0.46	2.71	−0.09	2.79	−2.01
90	1.74	0.66	2.15	0.31	2.16	0.37	2.19	−0.02	2.47	−0.54	2.70	−0.14	2.78	−2.13
100	1.73	0.67	2.13	0.33	2.15	0.34	2.18	−0.06	2.46	−0.62	2.68	−0.19	2.76	−2.25

### 3.3. Magnetic properties

**Figure 3.**
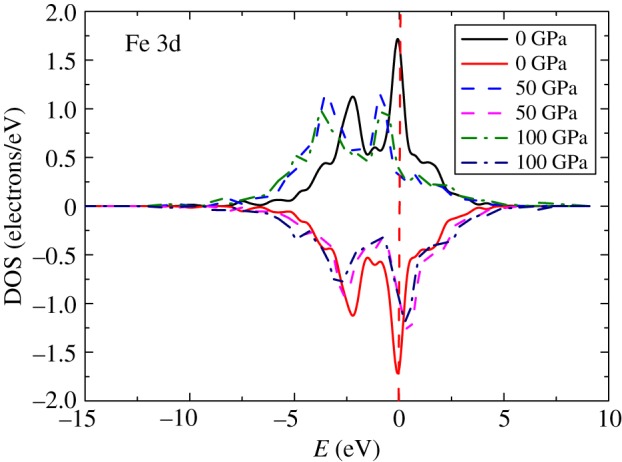
Spin-polarized DOS for Fe d bands of Mo_2_FeB_2_ under 0 GPa, 50 GPa and 100 GPa. The vertical dashed line at 0 eV is the Fermi level.

As the magnetic properties have significant impact on the crystal structures [11], the magnetic properties of the ground state should be decided. The calculated magnetic properties of Mo_2_FeB_2_ under different pressures are listed in table 3. In all cases, Mo_2_FeB_2_ shows AF behaviour. From the data in table 3, it can be found that the magnetic moments decrease with increasing pressure. The strong intra-band exchange interactions of the Fe d orbitals play a critical part in the magnetic moment of Mo_2_FeB_2_ [11]. There is no magnetic moment for Mo. Figure 3 illustrates the calculated Fe 3d DOS of Mo_2_FeB_2_ under the pressures of 0, 50 and 100 GPa. The examination of figure 3 also shows that the majority of spin channels are analogous to each other. Two obvious main peaks are present at 0 and −2.5 eV. With the pressure increasing, the heights of the peaks are reduced and the peaks move to lower energy, which is in good agreement with the decrease of the magnetic moment.

**Table 3.** Magnetic moment of Mo_2_FeB_2_ at different pressures.

	Fe (hbar/2)	
*P* (GPa)	up	down
0	2.04	−2.04
10	1.92	−1.92
20	1.81	−1.81
30	1.72	−1.72
40	1.63	−1.63
50	1.55	−1.55
60	1.47	−1.47
70	1.40	−1.40
80	1.33	−1.33
90	1.27	−1.27
100	1.21	−1.21

### 3.4. Elastic properties

Elastic constants depend on the stress and the strain tensors according to Hooke's law. The elastic constants *C_ijkl_* can be written as follows [23–25]:

3.1 $$C_{ijkl}={\left(\frac{\partial \sigma_{ij}(x)}{\partial e_{kl}}\right)}_{X},$$

where *e_kl_*, *σ_ij_*, *X* and *x* are, respectively, the Eulerian strain tensor, the applied stress tensor and the coordinates before and after the deformation. For the tetragonal crystal studied here, six independent elastic constants, *C*_11_, *C*_33_, *C*_44_, *C*_66_, *C*_12_ and *C*_13_, can be obtained. The bulk modulus (*B*) and the shear modulus (*G*) are deduced from the elastic constants. Based on the Voigt and Reuss method [26], for tetragonal crystals, the bulk modulus and the shear modulus are defined as

3.2 $$\begin{array}{rl} B_{V} & =\frac{2C_{11}+2C_{12}+4C_{13}+C_{33}}{9}, \end{array}$$

3.3 $$\begin{array}{rl} B_{R} & =\frac{1}{2(S_{11}+S_{12})+S_{33}+4S_{13}}, \end{array}$$

3.4 $$\begin{array}{rl} G_{V} & =\frac{2C_{11}-C_{12}-2C_{13}+C_{33}+6C_{44}+3C_{66}}{15} \end{array}$$

3.5 $$\begin{array}{rl} \text{and}\;G_{R} & =\frac{15}{8S_{11}-4S_{12}-8S_{13}+4S_{33}+6S_{44}+3S_{66}}. \end{array}$$

The arithmetic average of the Voigt and the Reuss bounds, which is the Voigt–Reuss–Hill (VRH) average, is considered to provide the best estimation of the isotropic elastic moduli [27]. Using the VRH average, the bulk and the shear modulus can be written as *B *= (*B*_V_ + *B*_R_)/2 and *G *= (*G*_V_ + *G*_R_)/2, respectively. The average *E* and Poisson's ratio (*v*) can be expressed with *B* and *G* as follows [28]:

3.6 $$\begin{array}{rl} E & =\frac{9BG}{3B+G} \end{array}$$

3.7 $$\begin{array}{rl} \text{and}\;\upsilon & =\frac{E-2G}{2G}. \end{array}$$

If the elastic constants satisfy the Born stability criterion, the crystal structure is usually considered to be mechanically stable [29,30]. A positive determinant for the crystal's symmetric matrix is required for the criterion of a stable crystal. For tetragonal crystals, the mechanical stability restrictions can be described as

3.8 $$\begin{array}{l} (C_{11}-P)>0,(C_{33}-P)>0,(C_{44}-P)>0,(C_{66}-P)>0,\\ (C_{11}-C_{12}-2P)>0,(C_{11}+C_{33}-2C_{13}-4P)>0,\\ (2C_{11}+2C_{12}+C_{33}+4C_{13}+3P)>0. \end{array}$$

**Figure 4.**
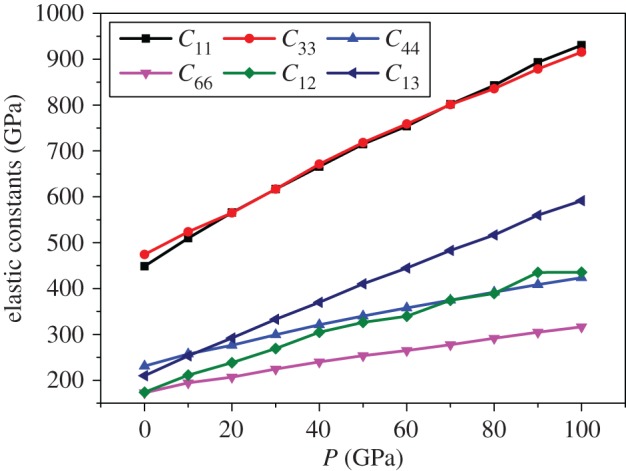
Pressure dependence of elastic constants of Mo_2_FeB_2_.

The elastic constants of Mo_2_FeB_2_ under different pressures are shown in figure 4. It was found that the elastic constants increase almost linearly with increasing pressure up to 100 GPa. This is caused by the enhancement of the covalent bonds (B–B, B–Fe) mentioned above. All elastic constants meet the Born stability criterion, indicating that Mo_2_FeB_2_ is mechanically stable from 0 to 100 GPa.

**Figure 5.**
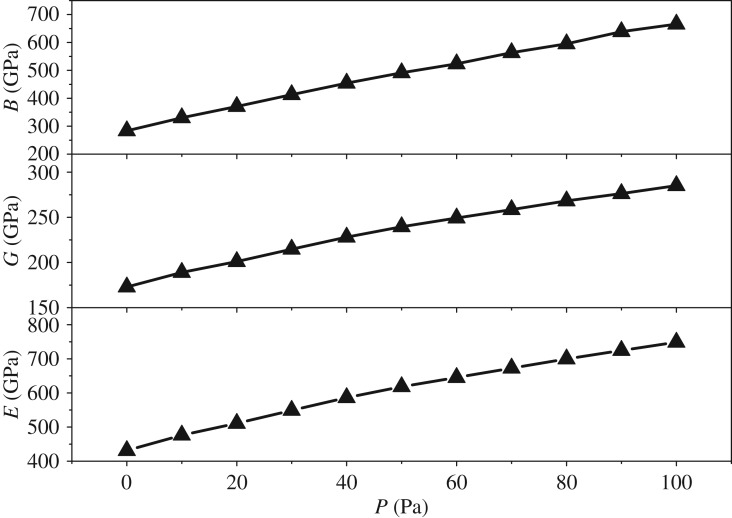
Pressure dependence of the bulk modulus, shear modulus and Young's modulus.

**Figure 6.**
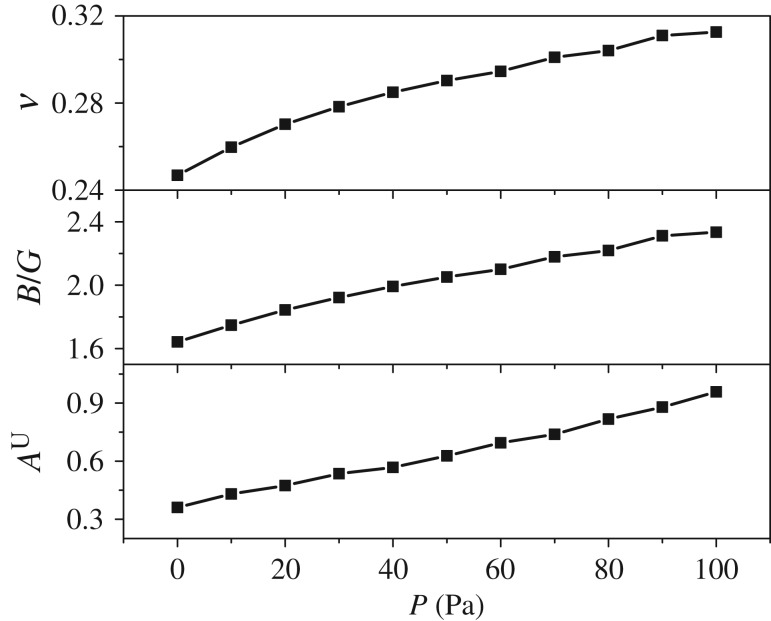
Calculated Poisson's ratio, the ratio of the bulk modulus to the shear modulus and the universal anisotropy index as functions of pressure.

Figure 5 shows a monotonic increase of *B* with the pressure. This finding means that the resistance ability of the material to uniform compression increases. *B* can also reflect the average atomic bond strength. Hence, the atomic bond strength of Mo_2_FeB_2_ increases with the pressure. Furthermore, the values of *G* and *C*_44_ of Mo_2_FeB_2_ also increase monotonically with the increase in pressure, thus indicating that it is harder to achieve a shear deformation with increasing pressure. The higher shear modulus implies the more pronounced directional interatomic bonding [31]. Thus, the bonding behaviour of Mo_2_FeB_2_ becomes more directional with the increase in pressure. Moreover, *E* also increases monotonically with the pressure, which means that it is harder to stretch the material uniformly with increasing pressure. As *B*, *G* and *E* increase monotonically with the pressure, the hardness is supposed to have a similar trend. Poisson's ratio, *B*/*G* and the universal anisotropy index (*A*^U^) are also calculated here, as shown in figure 6. Poisson's ratio is inversely proportional to the volume change during uniaxial deformation. That is, the lower the *υ* value, the larger is the volume change. The values of *υ* increase with the increase in pressure, thus indicating that there is lower volume change during uniaxial deformation. To analyse the ductile (brittle) behaviour of materials, a simple relationship has been proposed by Pugh: a high value of *B*/*G* corresponds to malleability, while a low value corresponds to brittleness [32]. It was suggested that 1.75 is the critical value that separates ductile and brittle materials. That is, if *B*/*G* > 1.75, the material behaves in a ductile manner. As shown in figure 6, Mo_2_FeB_2_ becomes more ductile as the pressure increases. When the pressure increases to 20 GPa, Mo_2_FeB_2_ changes from brittle to ductile. *A^U^* can be defined as

3.9 $$A^{U}=5\frac{G_{V}}{G_{R}}+\frac{B_{V}}{B_{R}}-6.$$

*A^U^* can indicate the degree of anisotropy for crystals. Zero means the crystal is isotropic. It is noted that *A^U^* increases with the pressure, which means that Mo_2_FeB_2_ is anisotropic under pressure.

**Figure 7.**
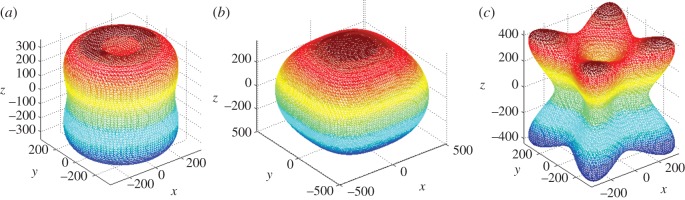
The directional-dependent Young's modulus of Mo_2_FeB_2_ under different pressures: (*a*) 0, (*b*) 50, (*c*) 100 GPa (the units are all in GPa).

In addition, the directional-dependent Young's modulus can also predict the elastic anisotropy of a crystal. For a tetragonal crystal this is expressed as follows [33]:

3.10 $$\frac{1}{E}=S_{11}({l_{1}}^{4}+{l_{2}}^{4})+(2S_{13}+S_{44})({l_{1}}^{2}{l_{3}}^{2}+{l_{2}}^{2}{l_{3}}^{2})+S_{33}{l_{3}}^{4}+(2S_{12}+S_{66}){l_{1}}^{2}{l_{2}}^{2},$$

where *l*_1_, *l*_2_ and *l*_3_ represent the directional cosines with respect to the *x*-, *y*- and *z*-axes, respectively. Using the compliance constant *S_ij_*, the directional Young's moduli for Mo_2_FeB_2_ were obtained, as shown in figure 7. For direct comparison, the directional-dependent Young's moduli are plotted in figure 7 for the pressures of 0, 50 and 100 GPa.

A spherical curved surface represents an isotropic system, while the deviation from the spherical shape indicates the extent of elastic anisotropy. For Mo_2_FeB_2_ at 0 GPa, the curved surface deviates slightly from the spherical shape, which means that there is a slight elastic anisotropy for Mo_2_FeB_2_, which is in agreement with the discussion above. When the pressure increased to 50 GPa, the curved surface changed to oval. This indicates that the elastic anisotropy increased. Furthermore, when the pressure reaches 100 GPa, the curved surface has a larger distortion, thus indicating larger anisotropy.

### 3.5. Thermal properties

To study the thermodynamic properties of Mo_2_FeB_2_ under high pressures, a quasi-harmonic Debye model [34] was used, in which the non-equilibrium Gibbs function G*(*V; p, T*) can be expressed as follows [35]:

3.11 $$G^{*}(V;p,T)=E(V)+pV+A_{\mathrm{vib}}[\Theta (V);T],$$

where *E*(*V*) is the total energy per unit cell, *pV* is the constant hydrostatic pressure condition, Θ(*V*) is the Debye temperature and *A*_vib_ is the vibrational term. *A*_vib_ can be expressed with the Debye model of the phonon DOS as follows [36]:

3.12 $$A_{\mathrm{vib}}(\Theta ;\;T)=nk_{B}T\left[\frac{9}{8}\frac{\Theta }{T}+3\ln(1-{\text{e}}^{-\Theta /T})-D\left(\frac{\Theta }{T}\right)\right].$$

Here, *n* is the number of atoms per formula unit, and *D*(Θ/*T*) represents the Debye integral. For an isotropic solid, Θ is defined as follows [34]:

3.13 $$\Theta =\frac{\hbar }{k_{B}}{\left[6\pi^{2}V^{1/2}n\right]}^{1/3}f(\sigma )\sqrt{\frac{B_{s}}{M}},$$

where *M* is the molecular mass per unit cell and *B*_s_ is the adiabatic bulk modulus, which is approximately given by the static compressibility [34]

3.14 $$B_{s}\approx B(V)=V\left(\frac{d^{2}E(V)}{dV^{2}}\right).$$

*f*(*σ*) is written as [37]

3.15 $$f(\sigma )={\left\{3{\left[2{\left(\frac{21+\sigma }{31-\sigma }\right)}^{3/2}+{\left(\frac{11+\sigma }{31-\sigma }\right)}^{3/2}\right]}^{-1}\right\}}^{1/3},$$

where *σ* is the Poisson's ratio. Thus, the thermal equation of state (EOS) *V(P,T)* can be calculated by the following equation with respect to volume *V*:

3.16 $${\left(\frac{\partial G^{*}(V;p,T)}{\partial V}\right)}_{p,T}=0.$$

The heat capacity *C_V_* and the thermal expansion coefficient *α* are defined as

3.17 $$\begin{array}{rl} C_{V,\text{vib}} & =3nk_{B}\left[4D\left(\frac{\Theta }{T}\right)-\frac{3\Theta /T}{e^{\Theta /T}-1}\right] \end{array}$$

3.18 $$\begin{array}{rl} \alpha & =\frac{\gamma C_{v}}{B_{T}V}, \end{array}$$

where *B*_T_ is the isothermal bulk modulus and *γ* is the Grüneisen parameter, which is expressed as

3.19 $$\gamma =-\frac{d\ln\Theta (V)}{d\ln V}.$$

**Figure 8.**
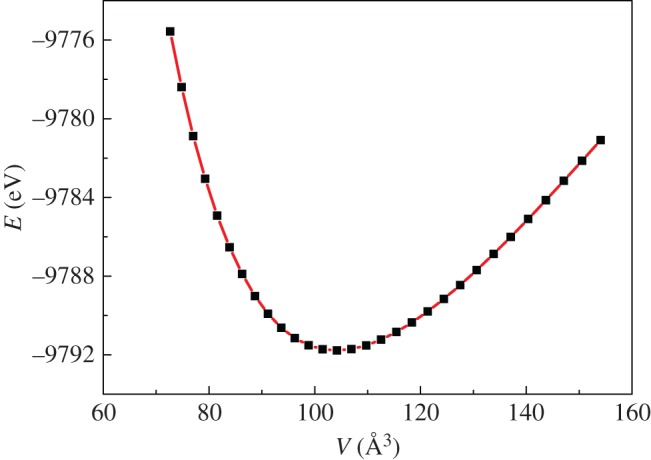
The correlation of the total energy (*E*) and unit cell (*V*) of Mo_2_FeB_2_. The black squares are the calculated values and the red line is the fitted curve with the Birch–Murnaghan EOS.

This paper calculated the pressure dependence of thermodynamic properties in the 0–100 GPa pressure range. First, a series of lattice constants were selected. Then, the corresponding unit cell volume and total energy were calculated and the third-order Birch–Murnaghan state equation was used for curve fitting to obtain the *E*–*V* curve (figure 8). As seen in the figure, the calculated values agreed well with the fitted values.

**Figure 9.**
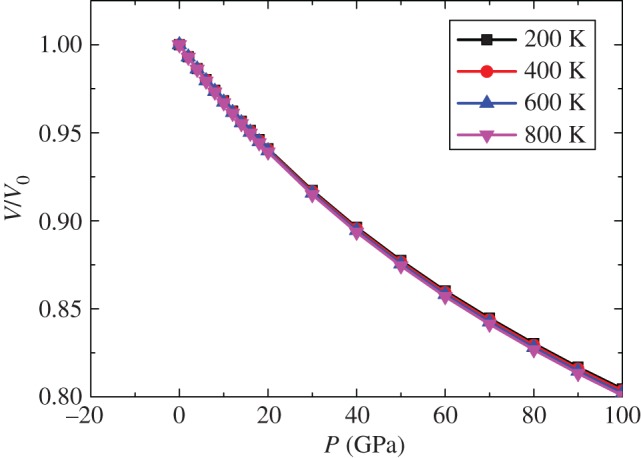
The normalized volume *V*/*V*_0_ as a function of pressure at temperature 200, 400, 600 and 800 K.

The dependence of the calculated normalized volume *V*/*V*_0_ on pressure *P* and temperature *T* is illustrated in figure 9, where *V*_0_ is the zero-pressure equilibrium volume. It is found that *V*/*V*_0_ decreases due to the increase in pressure and the slope of the curves also decreases, thus indicating that Mo_2_FeB_2_ is increasingly difficult to compress as the pressure increases. It is also found that the curves changed little with the increase in pressure, which means that Mo_2_FeB_2_ is stable under different temperatures.

**Figure 10.**
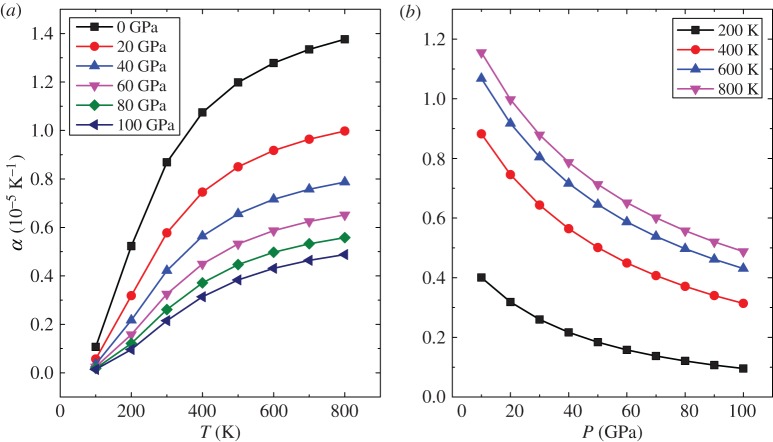
The thermal expansion as a function of temperature (*a*) and pressure (*b*).

The thermal expansion coefficient (*α*) can intuitively reflect a material's structural stability. Figure 10 shows the dependence of *α* on pressure and temperature. For a given pressure (figure 10*a*), *α* increases rapidly especially at zero pressure below a temperature of 400 K, and it increases slowly at higher temperatures. This is an expression of the excellent volume invariance under high temperature. However, *α* decreases strongly below 40 GPa with pressure at a constant temperature (figure 10*b*). Moreover, it decreases slowly above 40 GPa with the increase in pressure. This indicates that Mo_2_FeB_2_ possesses good volume invariance under high pressure.

**Figure 11.**
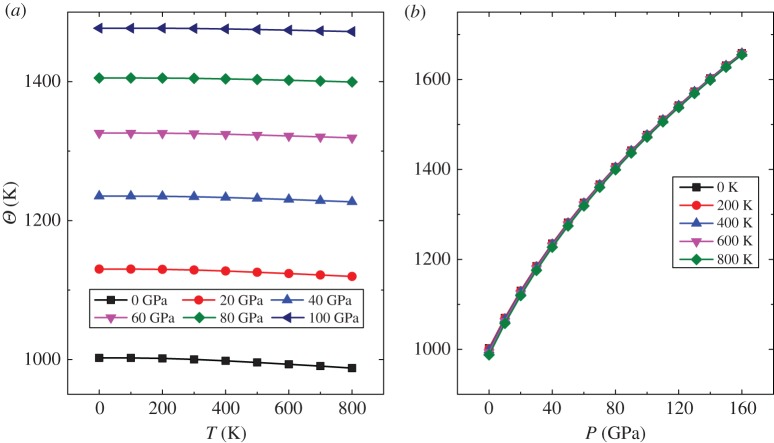
The Debye temperature as a function of temperature (*a*) and pressure (*b*).

Figure 11 shows the Debye temperature (*Θ*) as a function of the temperature and pressure. At room temperature (*T *= 300 K), *Θ* of 1000.19 K is obtained. Unfortunately, we did not find the corresponding experimental data. Under the application of pressure, *Θ* decreases very slowly with increase in temperature (figure 11*a*). Furthermore, at a given temperature (figure 11*b*), *Θ* tends to increase linearly with the increase in pressure. This indicated that the influence of the pressure on *Θ* is strong and that *Θ* is less affected by the temperature. The Debye temperature can also reflect the bonding between atoms. Thus, with the increase in pressure, the strength of atoms’ bonds increases, which is consistent with the above analysis.

**Figure 12.**
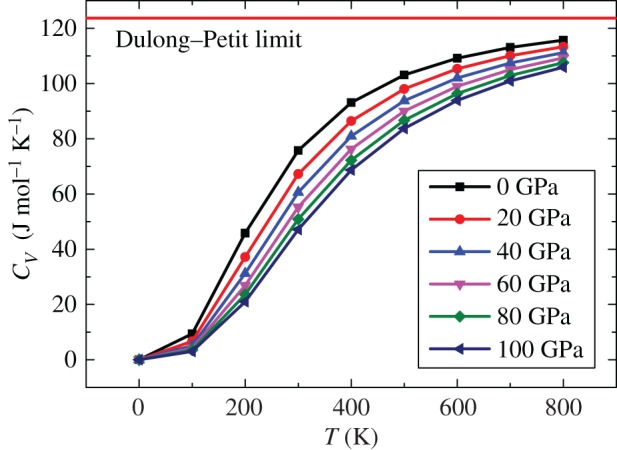
Temperature dependence of the heat capacity at different pressures.

Heat capacity *C_V_* is one of the most important parameters in thermodynamics. Figure 12 shows the relationship between the heat capacity and temperature under different pressures. For the same pressure, *C_V_* increases with the temperature. For the same temperature, *C_V_* decreases with the increase in pressure, thus implying that increasing the pressure is equivalent to reducing the temperature. The relationships of *C_V_* with the temperature and pressure show that *C_V_* is more sensitive to temperature than to the pressure. Owing to the anharmonic effect, when *T* < 500 K, the variation of *C_V_* with the changes in the temperature and pressure is more obvious. Under high temperature and high pressure, the heat capacity approaches the Dulong–Petit limit.

## 4. Conclusion

First-principles calculations were performed to investigate the structure, electronic DOS, and magnetic and elastic properties of Mo_2_FeB_2_ under high pressure. The volume of Mo_2_FeB_2_ decreases almost linearly with the increase in pressure. Examination of the DOS showed that with the pressure increasing, the B–B bonds were strengthened and the B–Mo covalency decreased. The atom population analysis and Mulliken overlap population analysis also found these results. With the pressure increasing, the sp hybridization of the B atoms increases, resulting in the increase of strong covalent bonding between the B atoms (forming a B–B bond). Moreover, the B–B and B–Fe bond populations increase with the increase in pressure, thus implying that the covalence of the B–B and B–Fe bonds increases. The analysis of the magnetic properties shows that, for all pressures, Mo_2_FeB_2_ shows AF behaviour and the magnetic moments decrease with the increase in pressure. The calculated *B*, *G*, *E*, *B*/*G*, *v* and *A^U^* all increase with the increase in pressure, which means that the hardness and ductility of Mo_2_FeB_2_ increase with the increase in pressure. Furthermore, from the directional-dependent Young's modulus of Mo_2_FeB_2_ under different pressures, it is found that elastic anisotropy increases with the increase in pressure. A quasi-harmonic Debye model was used to investigate the thermodynamic properties of Mo_2_FeB_2_ under high pressures. As the pressure increases, Mo_2_FeB_2_ is increasingly hard to compress. Furthermore, Mo_2_FeB_2_ is stable under different temperatures. From *α* analysis, it is found that Mo_2_FeB_2_ possesses good volume invariance under high pressure and temperature. The value of *Θ* is more influenced by pressure than by temperature. The examination of the relationships of *C_V_* with the temperature and pressure shows that *C_V_* is more sensitive to temperature than to pressure.

## Data accessibility

The data have been deposited at the Dryad Digital Repository (http://dx.doi.org/10.5061/dryad.8t70r) [38].
